# Suicidal and accidental drug poisoning mortality among older adults and working-age individuals in Spain between 2000 and 2018

**DOI:** 10.1186/s12877-022-02806-0

**Published:** 2022-02-10

**Authors:** Daniel Hernández-Calle, Gonzalo Martínez-Alés, Teresa López-Cuadrado

**Affiliations:** 1 La Paz University Hospital, Paseo de la Castellana, 261, 28046 Madrid, Madrid Spain; 2 Columbia University Mailman School of Public Health, New York, NY USA; 3 Mental Health Network Biomedical Research Center (CIBERSAM), Madrid, Spain; 4 Harvard University T.H. Chan School of Public Health, Boston, MA USA; 5 Hospital La Paz Institute for Health Research (IdiPAZ), Madrid, Spain; 6 National Epidemiology Center, Carlos III Health Institute, Madrid, Spain

**Keywords:** Geriatrics, Medication error, Intentional drug poisoning, Late-life suicide

## Abstract

**Background:**

Although medication poisoning in older adults is considered an increasingly important, but preventable cause of death, it has received relatively little attention. We explored recent trends and correlates of suicidal and accidental fatal drug poisonings among older and working-age individuals using nationwide data from Spain.

**Methods:**

We identified all 15,353 fatal drug poisonings involving decedents aged ≥15 years in Spain between 2000 and 2018 and divided them by age into older adults (≥65 years) and working-age (15-64 years) individuals. For each age group, we analyzed time trends in suicidal and accidental fatal drug poisoning rates (overall and by ICD-10 drug categories) using joinpoint regressions. To understand the specific drugs classified as “Non-psychotropic/non-specified”, we used 2018 data including substance-specific ICD-10 supplementary codes. We explored relevant sociodemographic correlates of suicidal and accidental fatal poisoning rates using multivariable negative binomial regressions.

**Results:**

Between 2000 and 2018, suicidal fatal poisonings increased faster among older (from 0.19 to 0.63 per 100,000 – average annual change: 7.7%) than working-age individuals (from 0.40 to 0.72 per 100,000 – average annual change: 3.8%). Accidental fatal poisonings increased among older adults (from 0.25 to 2.67 per 100,000 – average annual change: 16.2%) but decreased among working-age counterparts (from 2.38 to 1.42 per 100,000 – average annual change: − 1.9%). Anticoagulants and cardiac-stimulants glycosides accounted for 70% of the 223 accidental fatal poisonings due to non-psychotropic/non-specified drugs registered among older adults in 2018. Roles of gender and urban dwelling in suicidal and accidental poisonings were heterogeneous across age groups.

**Conclusion:**

Increases in suicidal drug poisonings were faster among older than working-age individuals. Accidental fatal poisonings increased only among older adults. Our findings that (i) sociodemographic correlates were heterogeneous across age groups and (ii) anticoagulant and cardiac-stimulant glycosides were particularly salient drivers of accidental poisonings among older adults have implications for prevention.

**Supplementary Information:**

The online version contains supplementary material available at 10.1186/s12877-022-02806-0.

## Introduction

Suicidal and accidental fatal drug poisonings among older adults constitute a major public health concern. Self-poisoning is the most common late-life suicide attempt method [1]. Moreover, suicidal self-poisoning among older people has shown a rising trend over the last years [2, 3]. Medication errors (ingestion of a wrong dose due to mistakes of the medication provider or the patient) are the most frequent cause of accidental poisoning in older adults [4]. Over the last decades, as medication regimes have become increasingly complex, the burden of health consequences and costs derived from medication errors among older adults has risen dramatically [5–7]. Accordingly, the World Health Organization has launched *Medication without harm,* an urgent call to improve prescribing, dispensing, and administration practices globally [8]. Illegal psychoactive drug overdose, traditionally considered an issue specific to younger ages, is increasingly salient among older adults, particularly due to the links between over-prescription practices and the ongoing opioid crisis [9, 10].

To reduce drug overdose mortality, it is critical to study accidental and suicidal poisonings together for various reasons. First, misclassification of fatal accidents as suicides and vice versa [11] is common, as determining the intentionality underlying a lethal overdose is often challenging or simply impossible [12]. Second, an important evidence-based prevention strategy for drug overdose mortality, regardless of the intentionality, is to restrict access to a small group of highly-toxic and widely available drugs that are responsible for a great proportion of overdose deaths [13]. For instance, substantial research links prescription practices to prescription and non-prescription opioid misuse and to both accidental and suicidal opioid overdose [9]. Third, even though suicidal and accidental poisonings are considered separate clinical entities, both share key actionable risk factors, such as polypharmacy, and cognitive impairment [14, 15].

Spain is one of the world’s most rapidly aging countries [16]. Reports highlight a significant recent rise in polypharmacy among older adults [17] - importantly, polypharmacy is a major preventable risk factor for accidental poisoning [8]. Along those lines, fatal medication errors occur in 0.5% of Spain’s hospital admissions constituting one of the highest fatal medication error rates in Europe [18]. In addition, increases in opioid use since 2000, especially among older adults [10], have risen concern regarding the onset of a potential opioid crisis across Europe [19]. Last, evidence suggests an ongoing upward trend of suicidal poisonings in adults in Spain [20]. However, no population-based studies have explored trends in accidental and intentional fatal drug poisonings in Spain, and fatal poisonings among older adults have not been examined, despite substantial implications for prevention efforts.

The goal of this study was to examine trends in accidental and suicidal fatal drug poisonings among older adults, overall and by specific drug involvement, comparing them to trends among working-age individuals, using Spain’s nationwide population-based mortality data between 2000 and 2018.

## Materials and methods

### Study setting, data source, and variables

We conducted a population-based study using data on all fatal drug poisonings registered in Spain between 1 January 2000 and 31 December 2018.

All data (mortality data and population data - used for the calculation of rates) come from the National Institute of Statistics. Mortality data are based on information from the National Mortality Registry and consist of single cause-of-death mortality statistics, extracted from death certificates based on the underlying cause-of-death and issued by medical examiners (following routine autopsy if criminality is suspected). These data follow ICD coding rules: the letter and first number specifies the intentionality ascribed to the poisoning (X4*: accidental, X6*: intentional, and Y1*: undetermined) and the second number specifies the drug involved using five categories (**0: nonopioid analgesics, antipyretics and antirheumatics, **1: antiepileptic, sedative-hypnotic, antiparkinsonism and psychotropic drugs, **2: narcotics and psychodysleptics, **3: other drugs acting on the autonomic nervous system, and **4: other and unspecified drugs, medicaments and biological substances) (codes **5-9 involve non-drug substances, such as alcohol or pesticides). For instance, an accidental methadone overdose would receive code X42. We selected decedents aged 15 or older whose ICD-10 underlying cause-of-death was an accidental, suicidal, or undetermined-intent poisoning, and grouped the drugs into two broader categories: Psychotropic drugs (X41, X42, X61, X62, Y11, and Y12) and Non-psychotropic/Non-specified drugs (X40, X43, X44, X60, X63, X64, Y10, Y13, and Y14).

In addition to the underlying cause of death, captured in traditional single cause-of-death mortality registries, death certificates provide information on additional contributing causes. This information is encoded using secondary ICD-10 codes in modern multiple cause-of-death mortality registries. For instance, specific substances are identified using ICD-10 “Poisonings by drugs, medicaments, and biological substances” T36-50 supplementary codes [21]. An accidental overdose by methadone would indicate, in addition to the underlying cause of death code X42 (Accidental poisoning by and exposure to narcotics and psychodysleptics), a contributing cause of death code T40.3 (i.e., poisoning by methadone). In Spain, multiple cause-of-death data became available in 2018. Accordingly, we used 2018 data to further understand the role of salient substances included under the Non-psychotropic/Non-specified category [22]. Because data for this study are completely de-identified, informed consent was not required - according to Spanish law.

In addition, we retrieved information on age, gender, and area of residence for each death.

### Analyses

Decedents were classified according to age into working-age individuals (15-64 years) or older adults (≥65 years). Area of residence was categorized into rural (< 10,000 inhabitants) or urban (≥10,000 inhabitants).

First, crude and age-adjusted mortality rates per 100,000 inhabitants were calculated using the standard European population as the reference population. We also estimated age group- and drug category-specific trends in accidental, suicidal, and undetermined-intent fatal poisonings.

Then, Joinpoint regression models were used to analyse temporal trends in fatal poisoning rates. This method uses generalized linear models, assuming a Poisson distribution, to identify inflection points (years), allowing for the quantification of temporal trends between consecutive inflection points (Annual Percent Changes [APC]), as well as over the entire time period (Average Annual Percent Changes [AAPC]), both including 95% confidence intervals [23, 24].

We sought to explore the possibility that accidental drug overdoses in working-age individuals may somewhat hide misclassified suicides [25]. Notably, in Spain, psychotropic drug poisonings involving use of narcotics and psychodysleptics not elsewhere specified (i.e., ICD-10 code X42) classified as accidental reliably represent true accidental overdoses, because data on these deaths are further collated by medical examiners [26]. Hence, we subtracted accidental drug poisonings including an ICD-10 code X42 from total accidental drug poisonings. Using the remaining number of drug poisonings, we estimated trends in potential misclassified suicidal overdoses over the study period and compared them to trends in drug poisonings officially classified as suicidal. Further information on ICD-10 coding rules for fatal poisonings is provided in Additional file 1.

To quantify the association between sociodemographic variables and the rate of accidental and suicidal fatal poisonings, we obtained overall and age group-specific incidence rate ratios, using multivariable negative binomial regression models given the overdispersion of the outcome.

For data analysis, we used STATA 16 (StataCorp College Station TX) and Joinpoint Regression program version 4.7.0.0.

## Results

### Number of fatal drug poisonings in the 2000-2018 period

Between 2000 and 2018, 15,353 fatal drug poisonings were registered in people aged 15 or older in Spain - 18.4% corresponding to older adults. This represents an annual 808 deaths, 5.3% of the total mortality due to external causes. The most frequent underlying causes of fatal drug poisoning were accidental (72.6%), suicidal (25.8%), and undetermined intent (1.6%). Fatal poisonings were most frequently attributed to non-psychotropic/non-specified drugs, accounting for 87.7% of deaths in older adults and 62.7% in working-age individuals.

### Mortality trends

During the study period, the age-adjusted rate of fatal poisonings remained relatively stable at around 2.3 per 100,000 (with slight decreasing and increasing trends, between 2000 and 2010 and 2011-2018, respectively) (Additional file 2, Fig. B.1), mainly because the rate of accidental poisonings remained roughly unchanged. Suicidal poisonings, however, increased an annual 4.1% (95% CI: 3.0, 5.3), from 0.35 per 100,000 in 2000 to 0.71 per 100,000 in 2018. Undetermined-intent poisonings rates were 0.05 per 100.000 in 2000 and decreased an annual 3.5% (95% CI: − 6.6, − 3.5) over the study period (A  dditional file 2, Fig. B.2).

**Fig. 1:**
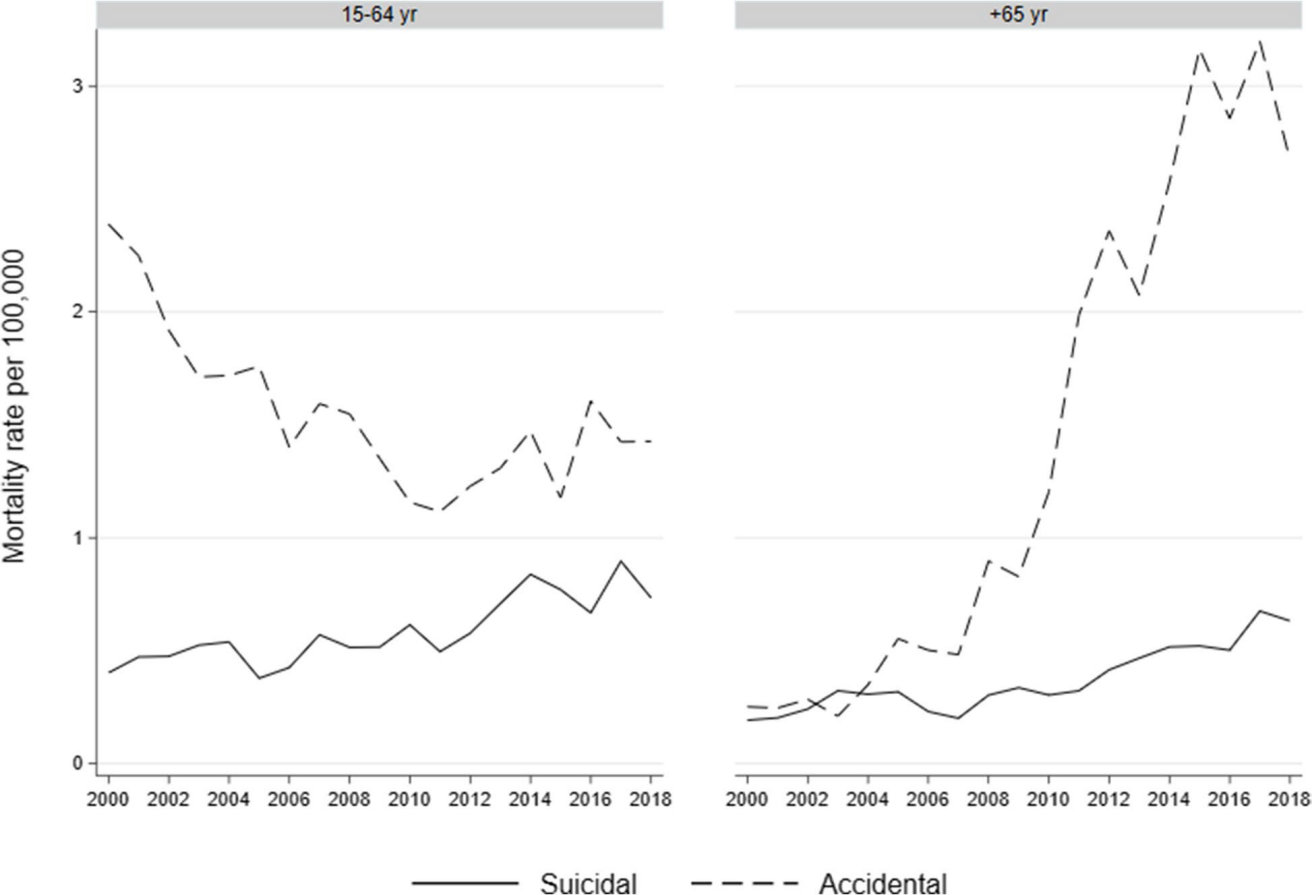
Crude mortality trends from suicidal and accidental poisonings in older and working-age adults

Notably, fatal poisoning rates were heterogeneous across age. Since crude and age-adjusted rates did not differ, we only present crude rates. As shown in Fig. 1, drug poisoning suicides increased from 0.19 per 100.000 in 2000 to 0.63 per 100.000 in 2018 among older adults - for an average annual 7.7% (95% CI: 2.3, 13.4) change; and from 0.40 per 100.000 in 2000 to 0.72 per 100.000 in 2018 among working-age individuals, for an average annual 3.8% (95% CI: 2.6, 5.1) change. Accidental poisonings increased from 0.25 per 100.000 in 2000 to 2.67 per 100.000 in 2018 among older adults, for an average annual 16.2% (95% CI: 12.2, 20.3) change; and decreased from 2.38 in 2000 to 1.42 in 2018 in working-age individuals, for an average annual − 1.9% (95% CI: − 0.4, − 3.5) change.

**Fig. 2:**
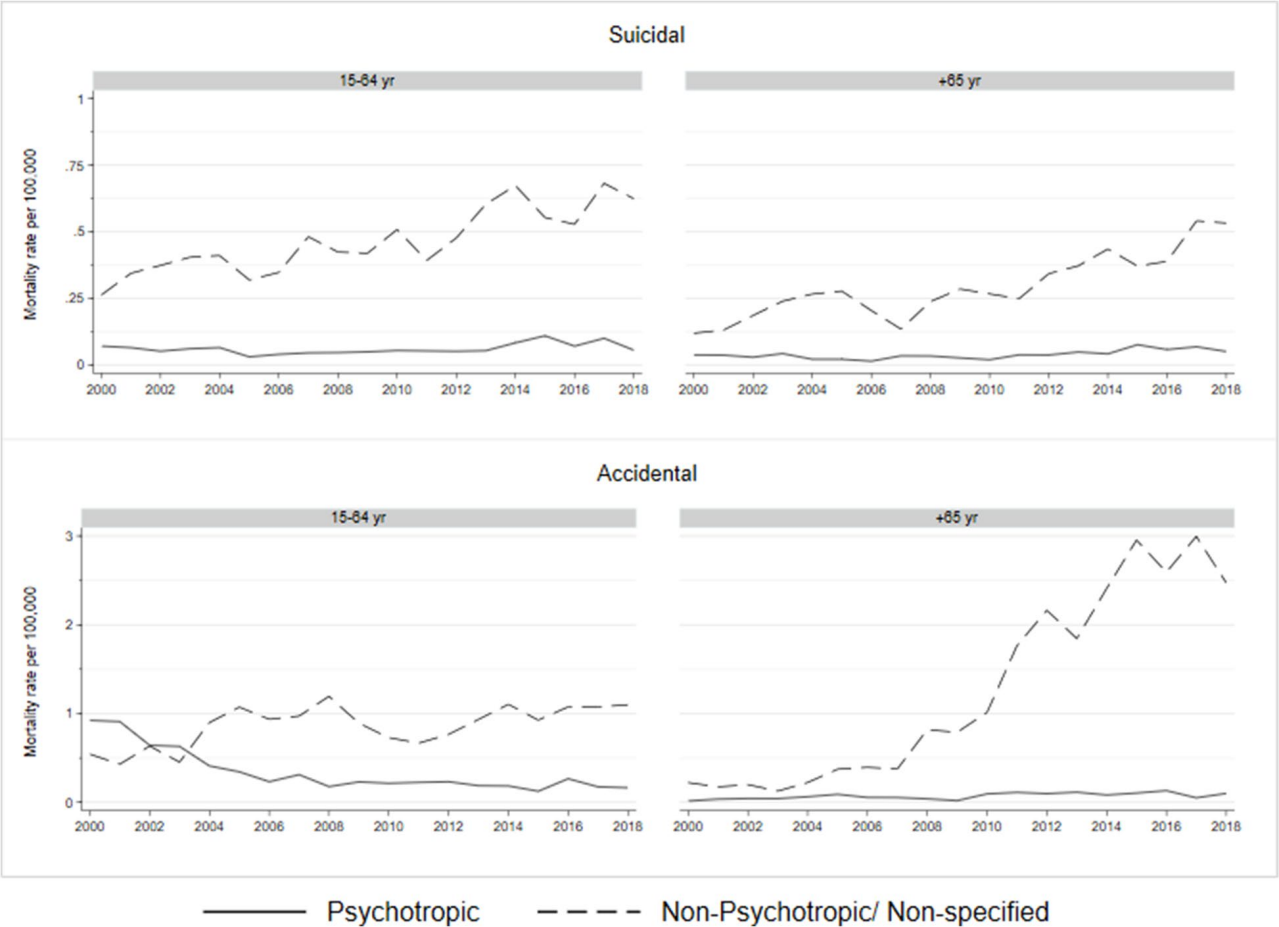
Crude mortality trends from suicidal and accidental poisonings by psychotropic and non-psychotropic/non-specified drugs in older and working-age adults

Poisonings by non-psychotropic/non-specified drugs drove the highest mortality rates in suicides in both age groups and in accidents in older adults (Fig. 2). Among working-age individuals, psychotropic drugs were initially the most salient drivers of accidental fatal poisonings, losing that position to non-psychotropic drugs after 2004 - mostly because accidental mortality due to psychotropic drugs decreased at an average annual − 17.5% (95%: − 21.2, − 13.6) between 2000 and 2008. Suicidal poisoning mortality rates due to psychoactive drugs did not experience changes over the study period.

**Fig. 3:**
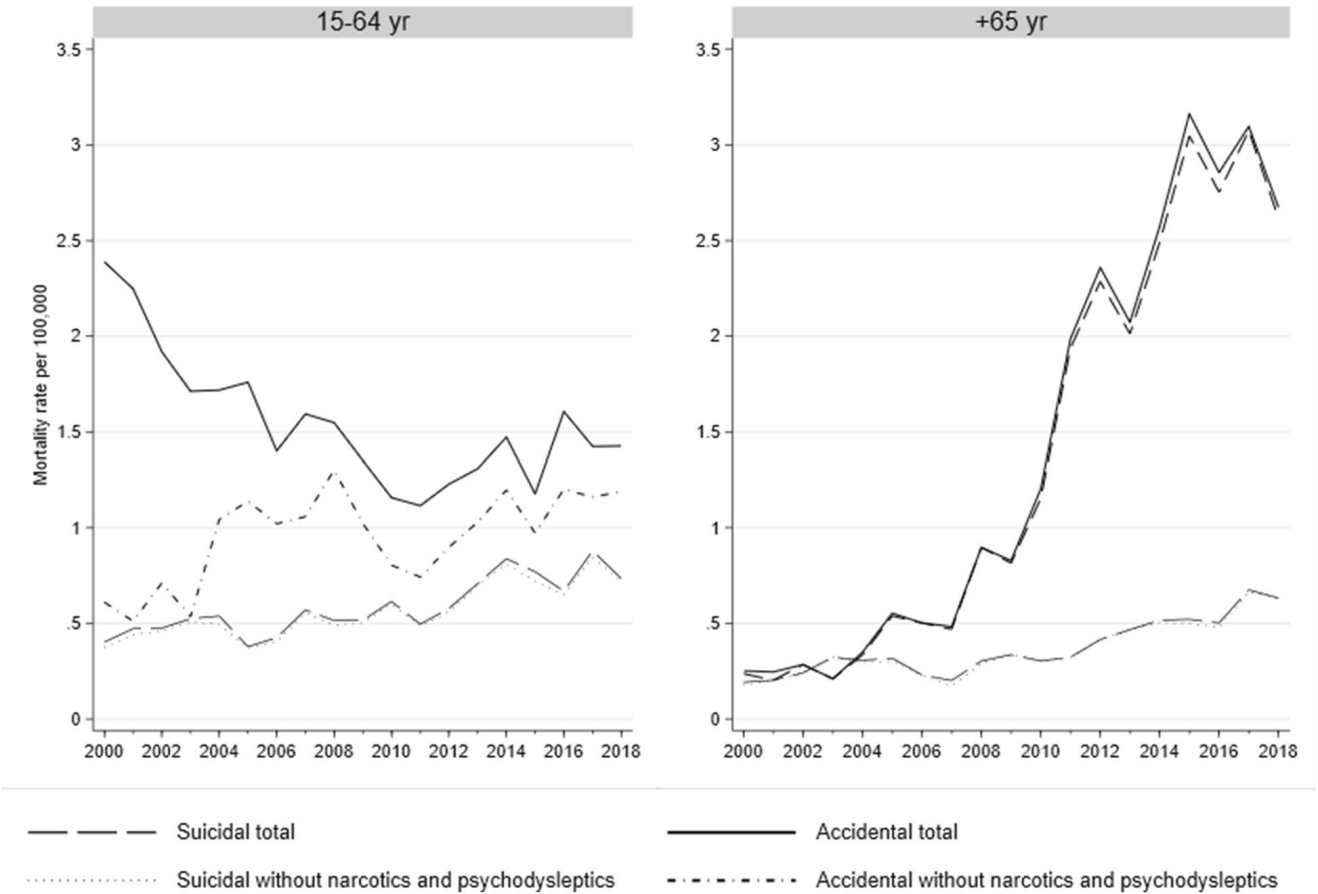
Mortality trends from suicidal and accidental drug poisonings by all drugs groups (total) and all drug groups after the subtraction of codes indicating narcotics and psychodysleptics

Figure 3 represents suicidal and accidental drug poisoning mortality rates after subtracting poisonings due to narcotics and psychodysleptics not elsewhere specified in both age groups. Among older adults, trends remained unchanged. Among working-age individuals, subtracting poisonings due to narcotics and psychodysleptics not elsewhere specified resulted on an average annual 4.2% (95% CI: 0.2, 8.5) increase in accidental poisonings and no change in suicidal poisonings.

### Sociodemographic characteristics of accidental and suicide fatal poisonings in the 2000-2018 period

After adjusting for sex, area of residence, and year, incidence of fatal drug poisonings was lower among older adults in comparison to younger counterparts for suicidal poisonings (Incidence Rate Ratio: 0.74; 95% CI: 0.66, 0.82) but not for accidental poisonings (IRR: 1.20; 95% CI: 0.91, 1.59).

Table 1 represents crude and adjusted IRR estimates of suicidal and accidental poisonings by sex, urbanicity, and year, stratified by age group. Among older adults, suicidal poisonings were 35% more frequent in urban areas compared to rural ones, showing no clear sex-specific variation; and accidental poisonings were 66% more frequent in rural areas and slightly more common among women. Among working-age individuals, while suicidal poisonings were 18% more frequent in rural areas, and 25% more frequent among men; accidental poisonings were not associated with urbanicity level but showed a remarkably gendered pattern: men had a 5-fold risk increase compared to women.

**Table 1:** Age-stratified unadjusted and adjusted negative binomial regressions models of accidental and suicidal poisonings mortality in older and working-age adults. All models are adjusted by study year

**Suicidal drug poisoning mortality**				
	IRR (95% CI) *p*-value			
	15-64 years		+ 65 years	
	Unadjusted	Adjusted	Unadjusted	Adjusted
**Sex**				
Women	Ref	Ref	Ref	Ref
Men	1.29 (1.12-1.50), *p*=0.001	1.25 (1.14-1.39), *p*<0.001	1.09 (0.85-1.41); *p*=0.49	1.08 (0.92-1.30); *p*=0.34
**Residence**				
Urban	Ref	Ref	Ref	Ref
Rural	1.19 (1.01-1.38); *p*=0.03	1.18 (1.06-1.32) *p*=0.003	0.62 (0.47-0.84) *p*=0.002	0.65 (0.50-0.83) *p*=0.01
**Accidental drug poisoning mortality**				
	IRR (95% CI) *p*-value			
	15-64 years		+ 65 years	
	Unadjusted	Adjusted	Unadjusted	Adjusted
**Sex**				
Women	Ref	Ref	Ref	Ref
Men	5.02 (4.45-5.68) *p*<0.001	5.02 (4.45-5.66) *p*<0.001	0.84 (0.54-1.30) *p*=0.43	0.88 (0.74-1.01) *p*=0.07
**Residence**				
Urban	Ref	Ref	Ref	Ref
Rural	0.93 (0.65-1.32) *p*=0.67	0.95 (0.84-1.08) *p*=0.44	1.65 (1.08-2.53) *p*=0.02	1.66 (1.51-1.83) *p*<0.001

### Specification of drugs included in the non-psychotropic/non-specified category using 2018 multiple cause-of-death data

**Fig. 4:**
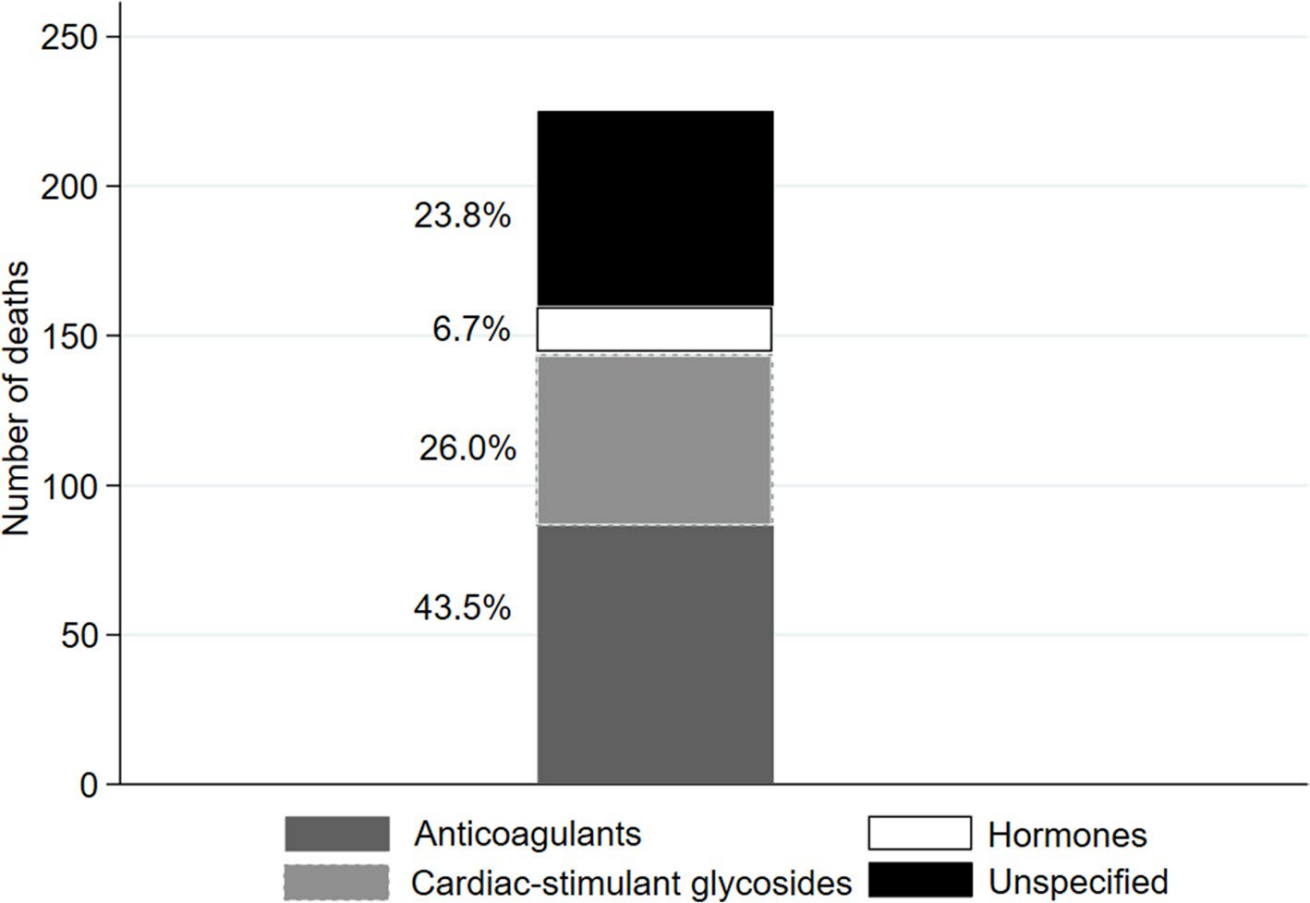
Number of deaths from accidental poisonings by detailed drug type in older adults in 2018. Total bar height represents number of deaths from agents from “Non-psychotropic/non-specified” drug category, while bar subdivisions represent cases from the specific drug categories available in multiple cause-of-death data. Percentages from bar subdivisions over total bar height are shown

Out of the 223 fatal accidental poisonings by non-psychotropic/non-specified drugs that occurred in older adults in 2018, multiple cause-of-death data allowed for the identification of specific drug codes for 76.2% of cases: 43.5% were due to anticoagulant drugs (ICD-10 Code: T45.5), 26.0% to cardiac-stimulant glycosides (ICD-10 Code: T46.0) and 6.7% to antidiabetic drugs (ICD-10 Code: T38.3) (Fig. 4). Supplementary codes were not available for most accidental poisonings involving working-age individuals, as well as for most suicides, caused by an agent included in the non-psychotropic/non-specified category. Accordingly, we could only identify specific drug codes for less than 2% of these deaths (Additional file 3, Figs. C.1 & C.2).

## Discussion

This is the first nationwide population-based study to examine trends in mortality due to drug poisoning in older adults. To ease interpretation, we compared fatal poisonings among older and working-age individuals. We analysed both suicidal and accidental poisonings because intentionality underlying fatal poisonings is often difficult to clarify [12] and, importantly, suicidal and accidental poisonings can both be prevented by limiting access to lethal substances [11]. Our results show that, between 2000 and 2018, trends in fatal drug poisonings were heterogeneous across age groups, with overall worse trends in older adults in comparison to working-age individuals. Among older adults, on average, accidental poisonings increased by an annual 16.2% (while they decreased among working-age individuals) and intentional poisonings increased by an annual 7.7% (roughly twice as much as among working-age individuals). These findings should generate substantial attention as they enhance our understanding of unnatural death among older adults and depict alarming trends, especially in the context of the global aging population.

Our finding that suicidal poisoning increased substantially among older adults and twice as fast as among working-age counterparts, even though overall suicide mortality among the Spanish geriatric population remained stable over the same period [27], does not lend itself to easy interpretation. As mentioned, access to lethal means is considered the most salient actionable predictor of death by suicide. Accordingly, it seems plausible that increased drug accessibility may have played an important role. Since most older adults who attempt suicide use their own prescribed medication [14], recent increases in drug prescription and polypharmacy among older adults [17] seem to support this possibility. Notably, depression may have played a crucial role as it is considered one of the most important risk factors for suicide, especially in older adults [28], and is also an independent risk factor for excessive polypharmacy [29]. Furthermore, antidepressants are one of the most common contributors of polypharmacy and, when present, they are the second most frequently interacting drug [30]. We detected higher poisoning suicide mortality among older adults living in urban rather than rural areas, a finding that could be linked to the overall higher prevalence of depression in urban environments [31]. It also seems plausible that this finding may be related to geographical differences between rural and urban areas in access to emergency medical care, an important determinant of outcomes following acute drug poisonings [2]. We should note that the increase in suicidal poisonings may also be partially explained by progressive improvements in mortality statistics, especially given that undetermined intent poisonings decreased over the same period.

Differences between suicidal behaviours among older and working-age individuals are crucial for prevention efforts. For instance, suicide attempts seem to entail higher lethality among older adults: while, overall, it takes adults around 20 suicide attempts to die by suicide, older adults die, on average, during their 4th suicide attempt [28]. Also, older adults show a lower tendency to consult with mental health providers during suicidal crises. Moreover, many suicide risk factors specific to older adults constitute barriers for help seeking, such as social disconnectedness – an increasingly frequent problem in this age group with substantial impact on mental health [32]), visual and hearing impairment, or walking difficulties [33]. As a result, several authors have emphasized the need of specific preventive strategies that tackle these difficulties, such as improving suicide risk and depression screening in general medical services, making mental health treatment facilities more age-friendly, and early prevention of social disconnectedness through social care evaluation [28, 34]. Even though suicide rates are higher in older adults, suicide prevention efforts have traditionally focused on other high risk populations (such as adolescents and young adults or people diagnosed with mental disorders), somewhat neglecting prevention of suicide among older adults [35].

Mortality due to accidental drug overdoses in older adults rose dramatically between 2000 and 2018. The low frequency of drug overdoses involving narcotics and psychodysleptics in this age group suggests that accidental deaths are mostly due to medication errors, including errors by both providers (prescription, administration, dispensing) and patients. The frequency of medication errors is high in Spain [36] and, as in other European countries, has been increasing over recent years [37]. It is worth mentioning that The Global Burden of Disease includes deaths coded as fatal accidental poisonings as illegal drug overdoses and not under the “Adverse effects of medical treatment” section, probably leading to an overall underestimation of estimates of mortality due to medication errors, which are especially frequent in older adults [38]. Incidence of and mortality due to medication errors has been the target of a solid body of investigation. Among the most salient risk factors identified among older adults, the following stand out: dementia, because of difficulties in adequately following medication regimes [15]; chronic kidney disease, which decreases the blood clearance of drugs [39]; and polypharmacy [8].

Preventive medication error strategies seek to bolster a culture of prevention that improves coordination between the patient and the different professionals involved in the prescription process (e.g., physician, nurse, pharmacist). Actions at several levels of healthcare planning can enhance prevention of medication errors. Since most accidental poisonings occur in the home [15, 40] and nursing homes [41], the most important prevention intervention consists on encouraging patient-provider and interprofessional coordination to improve adherence to the correct dosing regimen [8, 42, 43], as well as promoting social resources as teleassistance and domiciliary support when necessary [44]. Along these lines, improving access to healthcare is essential to ease patient-provider communication and further prevent medication errors. Accordingly, reducing formal (e.g., administrative) and informal (e.g., geographical) barriers is important for prevention purposes. In Spain, where a tax-funded health system provides universal healthcare to the population, efforts should preferentially target reducing informal differences in access to healthcare. Electronic administration systems suppose a major opportunity to improve medication reconciliation and clinical monitoring. Our results suggest that it would be particularly important to improve the implementation of electronic administration systems in rural areas, where we detected the highest mortality rate resulting from accidental drug poisonings and healthcare facilities are more scarcely distributed.

Simplifying medication regimes and limiting the prescription of a reduced group of highly toxic pharmacologic agents also should reduce mortality from poisoning in older adults, regardless of intentionality. Here, we found that roughly 9 in 10 fatal poisoning was due to drugs pertaining to the non-psychotropic/non-specified category; according to 2018 data, 43.5% corresponded with anticoagulant agents and 26.0% with cardiotonic glycosides. In Spain, the only widespread, highly toxic drugs from these categories are, respectively, acenocoumarol and digoxin – which have also been identified as key agents driving rates of severe medication errors elsewhere [5, 45]. Promoting use of safer pharmacological alternatives (such as so-called new oral anticoagulants beta blockers, or calcium channel blockers) when possible, may lead to a reduction in mortality from medication accidents. Our data did not allow for the specification of the drugs involved in suicidal poisonings. However, it has been reported that accidental and suicidal poisonings in this age group are frequently caused by the same drugs [1, 2, 14]. Therefore, it seems safe to conclude that a tighter control in the prescription of acenocumarol and digoxin may also reduce suicidal poisonings mortality rate. Additional measures, such as lethal means counselling or secure storing devices, also seem advisable to reduce suicidal lethal drug poisonings [46].

Working-age individuals had the highest accidental poisoning mortality rate, mainly due to a greater incidence of overdoses involving narcotics and psychodysleptics. In fact, mortality rate due to accidental poisonings exceeded the rate of suicidal poisonings in this age group. One possible explanation is that accidental fatal poisonings hide suicides to some extent, as previously suggested by other authors [25]. To further explore this possibility, we conducted a set of sensitivity analyses based on the rate of fatal accidental poisonings after excluding poisonings due to narcotics and psychodysleptics, which in Spain likely represent actually accidental overdoses, according to official sources [26]. Our finding that trends in deaths due to accidental poisonings not involving narcotics and psychodysleptics were markedly similar to trends in suicidal lethal poisonings supports this first hypothesis. A second possibility is that the non-psychotropic/non-specified category has been used in working-age individuals to encode accidental poisonings involving narcotics and psychodysleptics, something that has been suggested elsewhere [46]. Forensic records indicate that overdose mortality due to narcotics and psychodysleptics has remained stable over the last 20 years, lending support to this second hypothesis [26]. However, we should note that recreational and suicidal intentionality often coexist in overdoses involving drugs with dependence potential. Since preventive measures overlap, the difference between accidental and intentional overdoses may be simply a theoretical concept with limited intervention implications [47]. Notably, we found that psychotropic drugs mortality rate remained stable over the study period and was especially low among older adults, indicating absence of a current opioid epidemic in Spain as suggested by steady rates of opioid-related hospital emergencies [25].

### Limitations

These study findings should be interpreted in consideration of some limitations. First, while multiple cause-of-death mortality data provided useful insights regarding the specific drugs involved in accidental fatal poisonings in older adults in 2018, our conclusions are limited by lack of these data for previous years and suicidal poisonings. Further analyses, as more years of multiple cause-of-death mortality data become available, are warranted. Second, suicide trends to be underreported in mortality statistics as it tends to be misclassified and coded as undetermined-intent death [12]. This code, however, is infrequently used in Spanish data [46]. Nevertheless, two recent regional studies that compared registers from the National Institute of Statistics and forensic databases found data compatible with underreporting of accidental and suicidal poisonings [48, 49]. Accordingly, we cannot rule out some degree of underestimation of fatal poisonings. Third, we should acknowledge that older adults are not a homogeneous group. Notwithstanding, the limited number of suicidal and accidental poisonings among the very old (e.g., individuals aged 80 years and older) prevented us from estimating their mortality rates.

This study also has several strengths. As we used data from nationwide population-based registers, mortality rates and associations between risk factors and mortality risk should be considered valid and nationally representative. Also, the availability of a long time series ensured that mortality specific rates were not affected by changes in coding styles, a frequent bias in mortality studies based on deaths certificates [50].

## Conclusions

Drug poisoning is an increasingly important cause of death in older adults in Spain. Both suicidal and accidental poisonings mortality rates have significantly risen between 2000 and 2018 among older adults, at a higher rate than among working-age individuals. This finding highlights the importance of promoting age-specific suicide and medication error preventative strategies. For instance, as acenocoumarol and digoxin are seemingly responsible of a large proportion of drug poisonings in late life, strategies to reduce their prescription and increase safety of use may lead to substantive declines of mortality due to drug poisoning in this age group. Further improvements of the granularity and comprehensiveness of mortality data are warranted to enhance understanding and prevention of drug poisoning overall and among older adults, an especially vulnerable population.

## Supplementary Information

**Additional file 1.**

**Additional file 2.**

**Additional file 3.**

## Data Availability

The data that support the findings of this study are available from National Institute of Statistics (www.ine.es) but restrictions apply to the availability of these data, which were used under license for the current study, and so are not publicly available. Data are however available from the authors upon reasonable request and with permission of National Institute of Statistics.

## Abbreviations

ICD: International Classification of Diseases
APC: Annual Percent Changes
AAPC: Average Annual Percent Changes
IRR: Incidence Rate Ratio
