# Supplementary material for: Suicidal and accidental drug poisoning mortality among older adults and working-age individuals in Spain between 2000 and 2018
Source: BMC Geriatr. 2022 Feb 10;22:114. doi: 10.1186/s12877-022-02806-0 (PMC8832785; doi:10.1186/s12877-022-02806-0)
Supplement: Supplementary file 1 — Additional file 1. [file 12877_2022_2806_MOESM1_ESM.docx]

**Additional file 1:** Fatal poisonings ICD-10 coding rules.

ICD-10 rules state that in fatal poisonings the fundamental cause will be the code of accidental (X40-44), intentional (X60-64) or undetermined-intent (Y10-14) poisoning, included in Chapter XX. [External causes of morbidity and mortality (V01-Y98)] (Brämer, 1988). These codes have two parts:

1. Letter and first number: (eg. X4 from X40). They represent the intentionality of the poisoning (accidental X4*, intentional X6* or undetermined Y1*).

2. Last number: (eg. 0 from 40). It represents the drug involved, only allowing five drug categories as is it showed in the table 1A.

1.1.2 Multiple cause-of-death coding:

Multiple cause-of-death statistics allow the addition of a supplementary code (T36-T50) from Chapter XIX [Injury, poisoning and certain other consequences of external causes (S00-T98)]. These codes include 139 drug categories in which the coder can specify the drug involved (Table 2A.)

Multiple cause-of-death statistics require automate death certificate processing and coding. This technology was first implemented in the United States of America in 1970s and has been extended to other countries in the last two decades. The European Union developed its own technology in the 2000s, the software IRIS, that it is currently used in several European countries as well as other countries as Australia, Japan and Mexico. Multiple cause-of-death data became available in Spain in 2016 after implementing this software (Iris Institute).

**Table A1.** Chapter XX [External causes of morbidity and mortality] used in fatal poisonings.

X40/X60/Y10 Accidental / Intentional / Undetermined intent poisoning by and exposure to nonopioid analgesics, antipyretics and antirheumatics

4-aminophenol derivatives

nonsteroidal anti-inflammatory drugs [NSAID]

pyrazolone derivatives

Salicylates

X41/X61/Y11 Accidental / Intentional / Undetermined intent poisoning by and exposure to antiepileptic, sedative-hypnotic, antiparkinsonism and psychotropic drugs, not elsewhere classified

Antidepressants

Barbiturates

hydantoin derivatives

Iminostilbenes

methaqualone compounds

Neuroleptics

Psychostimulants

succinimides and oxazolidinediones

Tranquillizers

X42/X62/Y12 Accidental / Intentional / Undetermined intent poisoning by and exposure to narcotics and psychodysleptics [hallucinogens], not elsewhere classified

cannabis (derivatives)

Cocaine

Codeine

Heroin

lysergide [LSD]

Mescaline

Methadone

Morphine

opium (alkaloids)

X43/X63/Y13 Accidental / Intentional / Undetermined intent poisoning by and exposure to other drugs acting on the autonomic nervous system

parasympatholytics [anticholinergics and antimuscarinics] and spasmolytics

parasympathomimetics [cholinergics]

sympatholytics [antiadrenergics]

sympathomimetics [adrenergics]

X44/X64/Y14 Accidental / Intentional / Undetermined intent poisoning by and exposure to other and unspecified drugs, medicaments and biological substances

agents primarily acting on smooth and skeletal muscles and the respiratory system

anaesthetics (general)(local)

drugs affecting the:

cardiovascular system

gastrointestinal system

hormones and synthetic substitutes

systemic and haematological agents

systemic antibiotics and other anti-infectives

therapeutic gases

topical preparations

vaccines

water-balance agents and drugs affecting mineral and uric acid metabolism

anaesthetics (general)(local)

drugs affecting the:

cardiovascular system

gastrointestinal system

hormones and synthetic substitutes

systemic and haematological agents

systemic antibiotics and other anti-infectives

therapeutic gases

topical preparations

Vaccines

water-balance agents and drugs affecting mineral and uric acid metabolism

unespecified drugs

X85 Assault by drugs, medicaments and biological substances

homicidal poisoning by (any):

biological substance

Drug

Medicament

Table A.2: Supplementary codes from Chapter XIX [Injury, poisoning and certain other consequences of external causes] used in fatal poisonings.

ICD-10 code Description

T36 Poisoning by systemic antibiotics

T36.0 Poisoning by penicillins

T36.1 Poisoning by cephalosporins and other beta-lactam antibiotics

T36.2 Poisoning by chloramphenicol group

T36.3 Poisoning by macrolides

T36.4 Poisoning by tetracyclines

T36.5 Poisoning by aminoglycosides

T36.6 Poisoning by rifampicins

T36.7 Poisoning by antifungal antibiotics

T36.8 Poisoning by other systemic antibiotics

T36.9 Poisoning by unspecified systemic antibiotic

T37 Poisoning by other systemic anti-infectives and antiparasitics

T37.0 Poisoning by sulfonamides

T37.1 Poisoning by antimycobacterial drugs

T37.2 Poisoning by antimalarials and drugs acting on other blood protozoa

T37.3 Poisoning by other antiprotozoal drugs

T37.4 Poisoning by anthelminthics

T37.5 Poisoning by antiviral drugs

T37.8 Poisoning by other specified systemic anti-infectives and antiparasitics

T37.9 Poisoning by unspecified systemic anti-infective and antiparasitics

T38 Poisoning by hormones and their synthetic substitutes and antagonists, not elsewhere classified

T38.0 Poisoning by glucocorticoids and synthetic analogues

T38.1 Poisoning by thyroid hormones and substitutes

T38.2 Poisoning by antithyroid drugs

T38.3 Poisoning by insulin and oral hypoglycemic [antidiabetic] drugs

T38.4 Poisoning by oral contraceptives

T38.5 Poisoning by other estrogens and progestogens

T38.6 Poisoning by antigonadotrophins

T38.7 Poisoning by androgens and anabolic congeners

T38.8 Poisoning by unspecified hormones and synthetic substitutes

T38.9 Poisoning by unspecified hormone antagonists

T39 Poisoning by nonopioid analgesics, antipyretics and antirheumatics

T39.0 Poisoning by aspirin

T39.1 Poisoning by 4-aminophenol derivatives

T39.2 Poisoning by pyrazolone derivatives

T39.3 Poisoning by propionic acid derivatives

T39.4 Poisoning by antirheumatics

T39.8 Poisoning by other nonopioid analgesics and antipyretics

T39.9 Poisoning by unspecified nonopioid analgesic

T40 Poisoning by narcotics and psychodysleptics [hallucinogens]

T40.0 Poisoning by opium

T40.1 Poisoning by heroin

T40.2 Poisoning by other opioids

T40.3 Poisoning by methadone

T40.4 Poisoning by other synthetic narcotics

T40.5 Poisoning by cocaine

T40.6 Poisoning by unspecified narcotics

T40.7 Poisoning by cannabis (derivatives)

T40.8 Poisoning by lysergide [lsd]

T40.9 Poisoning by unspecified psychodysleptics [hallucinogens]

T41 Poisoning by anaesthetics and therapeutic gases

T41.0 Poisoning by inhaled anesthetics

T41.1 Poisoning by intravenous anesthetics

T41.2 Poisoning by unspecified general anesthetics

T41.3 Poisoning by local anesthetics

T41.4 Poisoning by unspecified anesthetic

T41.5 Poisoning by therapeutic gases

T42 Poisoning by antiepileptic, sedative-hypnotic and antiparkinsonism drugs

T42.0 Poisoning by hydantoin derivatives

T42.1 Poisoning by iminostilbenes

T42.2 Poisoning by succinimides and oxazolidinediones

T42.3 Poisoning by barbiturates

T42.4 Poisoning by benzodiazepines

T42.5 Poisoning by mixed antiepileptics

T42.6 Poisoning by other antiepileptic and sedative-hypnotic drugs

T42.7 Poisoning by unspecified antiepileptic and sedative-hypnotic drugs

T42.8 Poisoning by antiparkinsonism drugs and other central muscle-tone depressants

T43 Poisoning by psychotropic drugs, not elsewhere classified

T43.0 Poisoning by tricyclic and tetracyclic antidepressants

T43.1 Poisoning by monoamine-oxidase-inhibitor antidepressants

T43.2 Poisoning by unspecified antidepressants

T43.3 Poisoning by phenothiazine antipsychotics and neuroleptics

T43.4 Poisoning by butyrophenone and thiothixene neuroleptics

T43.5 Poisoning by unspecified antipsychotics and neuroleptics

T43.6 Poisoning by psychostimulants with abuse potential

T43.8 Poisoning by other psychotropic drugs

T43.9 Poisoning by unspecified psychotropic drug

T44 Poisoning by drugs primarily affecting the autonomic nervous system

T44.0 Poisoning by anticholinesterase agents

T44.1 Poisoning by other parasympathomimetics [cholinergics]

T44.2 Poisoning by ganglionic blocking drugs

T44.3 Poisoning by other parasympatholytics [anticholinergics and antimuscarinics] and spasmolytics

T44.4 Poisoning by predominantly alpha-adrenoreceptor agonists

T44.5 Poisoning by predominantly beta-adrenoreceptor agonists

T44.6 Poisoning by alpha-adrenoreceptor antagonists

T44.7 Poisoning by beta-adrenoreceptor antagonists

T44.8 Poisoning by centrally-acting and adrenergic-neuron- blocking agents

T44.9 Poisoning by unspecified drugs primarily affecting the autonomic nervous system

T45 Poisoning by primarily systemic and haematological agents, not elsewhere classified

T45.0 Poisoning by antiallergic and antiemetic drugs

T45.1 Poisoning by antineoplastic and immunosuppressive drugs

T45.2 Poisoning by vitamins

T45.3 Poisoning by enzymes

T45.4 Poisoning by iron and its compounds

T45.5 Poisoning by anticoagulants

T45.6 Poisoning by unspecified fibrinolysis-affecting drugs

T45.7 Poisoning by anticoagulant antagonists

T45.8 Poisoning by other primarily systemic and hematological agents

T45.9 Poisoning by unspecified primarily systemic and hematological agent

T46 Poisoning by agents primarily affecting the cardiovascular system

T46.0 Poisoning by cardiac-stimulant glycosides and drugs of similar action

T46.1 Poisoning by calcium-channel blockers

T46.2 Poisoning by other antidysrhythmic drugs

T46.3 Poisoning by coronary vasodilators

T46.4 Poisoning by angiotensin-converting-enzyme inhibitors

T46.5 Poisoning by other antihypertensive drugs

T46.6 Poisoning by antihyperlipidemic and antiarteriosclerotic drugs

T46.7 Poisoning by peripheral vasodilators

T46.8 Poisoning by antivaricose drugs

T46.9 Poisoning by unspecified agents primarily affecting the cardiovascular system

T47 Poisoning by agents primarily affecting the gastrointestinal system

T47.0 Poisoning by histamine h2-receptor blockers

T47.1 Poisoning by other antacids and anti-gastric-secretion drugs

T47.2 Poisoning by stimulant laxatives

T47.3 Poisoning by saline and osmotic laxatives

T47.4 Poisoning by other laxatives

T47.5 Poisoning by digestants

T47.6 Poisoning by antidiarrheal drugs

T47.7 Poisoning by emetics

T47.8 Poisoning by other agents primarily affecting gastrointestinal system

T47.9 Poisoning by unspecified agents primarily affecting the gastrointestinal system

T48 Poisoning by agents primarily acting on smooth and skeletal muscles and the respiratory system

T48.0 Poisoning by oxytocic drugs

T48.1 Poisoning by skeletal muscle relaxants [neuromuscular blocking agents]

T48.2 Poisoning by unspecified drugs acting on muscles

T48.3 Poisoning by antitussives

T48.4 Poisoning by expectorants

T48.5 Poisoning by other anti-common-cold drugs

T48.6 Poisoning by antiasthmatics

T48.9 Poisoning by unspecified agents primarily acting on the respiratory system

T49 Poisoning by topical agents primarily affecting skin and mucous membrane and by ophthalmological, otorhinolaryngological and dental drugs

T49.0 Poisoning by local antifungal

T49.1 Poisoning by antipruritics

T49.2 Poisoning by local astringents and local detergents

T49.3 Poisoning by emollients

T49.4 Poisoning by keratolytics

T49.5 Poisoning by ophthalmological drugs and preparations

T49.6 Poisoning by otorhinolaryngological drugs and preparations

T49.7 Poisoning by dental drugs

T49.8 Poisoning by other topical agents

T49.9 Poisoning by unspecified topical agent

T50 Poisoning by diuretics and other and unspecified drugs, medicaments and biological substances

T50.0 Poisoning by mineralocorticoids and their antagonists

T50.1 Poisoning by loop [high-ceiling] diuretics

T50.2 Poisoning by carbonic-anhydrase inhibitors

T50.3 Poisoning by electrolytic

T50.4 Poisoning by drugs affecting uric acid metabolism

T50.5 Poisoning by appetite depressants

T50.6 Poisoning by antidotes and chelating agents

T50.7 Poisoning by analeptics and opioid receptor antagonists

T50.8 Poisoning by diagnostic agents

T50.9 Other and unspecified drugs, medicaments and biological substances

References:

Brämer, G. R. (1988). International statistical classification of diseases and related health problems. Tenth revision. *World health statistics quarterly.* *41*(1), 32-36.

Iris Insitute (<https://www.dimdi.de/dynamic/en/classifications/iris-institute/#about-iris>) [Accessed 1/4/2021].
