# Supplementary material for: Suicidal and accidental drug poisoning mortality among older adults and working-age individuals in Spain between 2000 and 2018
Source: BMC Geriatr. 2022 Feb 10;22:114. doi: 10.1186/s12877-022-02806-0 (PMC8832785; doi:10.1186/s12877-022-02806-0)
Supplement: Supplementary file 2 — Additional file 2. [file 12877_2022_2806_MOESM2_ESM.docx]

**Additional file 2:** Age-adjusted mortality rates from drug poisonings in Spain between 2000 and 2018.

**Figure B.1**: Age-adjusted mortality rates from drug poisonings in Spain between 2000 and 2018.

**Figure B.2**: Age-adjusted mortality rates from accidental, suicidal and undetermined-intent drug poisonings in Spain between 2000 and 2018.
