# Supplementary material for: Suicidal and accidental drug poisoning mortality among older adults and working-age individuals in Spain between 2000 and 2018
Source: BMC Geriatr. 2022 Feb 10;22:114. doi: 10.1186/s12877-022-02806-0 (PMC8832785; doi:10.1186/s12877-022-02806-0)
Supplement: Supplementary file 3 — Additional file 3. [file 12877_2022_2806_MOESM3_ESM.docx]

**Additional file 3:** Drug specification in 2018 using multiple-cause-of-death data of accidental deaths and suicides in both age-groups.

**Figure C1.** Drug specification in 2018 using multiple-cause-of-death data of accidental deaths and suicidal poisonings in older.


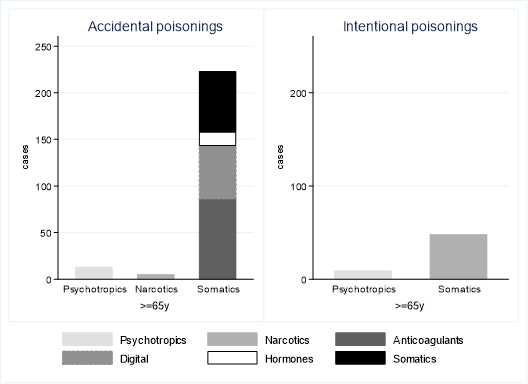


*NP/NS: Non-psychotropic/Non-specified drug category

**Figure C2.** Drug specification in 2018 using multiple-cause-of-death data of accidental deaths and suicidal poisonings in working-age adults.


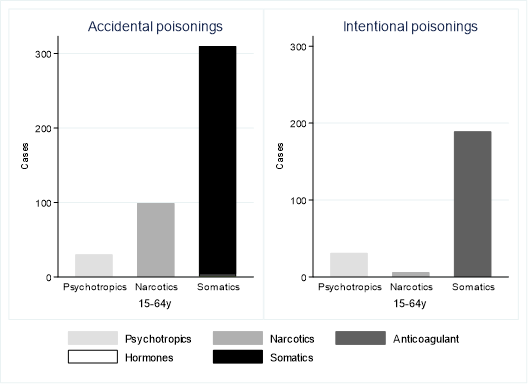


*NP/NS: Non-psychotropic/Non-specified drug category
